# The right ventricle outflow tract systolic function could predict the severity of the cirrhosis

**DOI:** 10.55730/1300-0144.5785

**Published:** 2023-11-11

**Authors:** İbrahim DÖNMEZ, Güray CAN, Emrah ACAR

**Affiliations:** 1 Department of Cardiology, Faculty of Medicine, Bolu Abant İzzet Baysal University, Bolu, Turkiye; 2 Department of Gastroenterology, Faculty of Medicine, Bolu Abant İzzet Baysal University, Bolu, Turkiye

**Keywords:** Child-Turcotte-Pugh, cirrhosis, echocardiography, outflow tract, right ventricle, systole

## Abstract

**Background/aim:**

The distinctive liver framework is converted into structurally abnormal nodules as a consequence of tissue fibrosis in cirrhosis. Cardiac dysfunction in cirrhosis was described, and the term “cirrhotic cardiomyopathy (CCM)” was coined to describe this syndrome. Recent research has shown that the contractile characteristics of the right ventricular outflow tract (RVOT) have a significant impact on right ventricular functions. The right ventricular outflow tract-systolic excursion is an important systolic function marker of RVOT (RVOT-SE). There has yet to be published research on RVOT function in cirrhotic patients. We looked at the relationship between cirrhosis severity and the RVOT-SE.

**Materials and methods:**

Sixty-nine consecutive hepatic cirrhotic patients were recruited for the research between June 1, 2018 and January 1, 2022. A medical history, thorough physical examination, laboratory investigations, echocardiographic evaluation, and RVOT-SE were obtained. The patients were separated into two groups: those with compensated cirrhosis (Child-Pugh class 1) and those with decompensated cirrhosis (Child-Pugh class 2 and 3).

**Results:**

On the numerous standard echocardiographic parameters that examined the diameter and function of the left ventricle, we observed no significant difference between groups. Nevertheless, a statistically significant difference in Right Ventricle Wall (RVW) (p = 0.014), systolic pulmonary artery pressure (sPAP) (p = 0.034), RVOT-SE (p = 0.003), and Tricuspid Annular Plane Systolic Excursion (TAPSE) (p = 0.042) was detected across cirrhosis groups. The RVOT-SE had a positive correlation with cirrhosis severity (OR: 0.607; 95% CI: 0.425–0.866; p = 0.006), according to multiple logistic regression studies. According to the ROC curve study, RVOT-SE 8.8 cm/s predicted decompensated cirrhosis with 72% sensitivity and 72.7% specificity (AUC = 0.715, p: 0.001).

**Conclusion:**

In the current study, we found that RVOT-SE was related to the severity of cirrhosis. Larger patient cohorts and multi-center investigations will provide light on the notion.

## 1. Introduction

Cirrhosis is often triggered by the alteration of normal liver architecture into anatomically disordered nodules as a consequence of tissue fibrosis [1]. Cirrhosis and chronic liver disease (CLD) are major sources of morbidity and death, despite the fact that their burden and underlying causes differ across the globe [2]. Cirrhotic patient outcome prediction and overall mortality risk calculation are critical therapeutic tasks. As a result, many scores for measuring short- and long-term mortality, as well as the etiology of chronic liver disease, have been established and recommended. The Child-Turcotte-Pugh score, one of the most verified and extensively used scoring systems, is simple to compute and appropriate for a variety of etiologies of liver disease [3].

Cardiac dysfunction in cirrhosis was originally documented five decades ago, and the name “cirrhotic cardiomyopathy (CCM)” for this disease was coined in 1989 [4]. CCM formation is complicated and depends on systemic alterations caused by underlying cirrhosis. Cirrhosis-related cardiovascular problems include lower mean arterial pressure, decreased peripheral vascular resistance, and enhanced cardiac output; this combination is known as “hyperdynamic circulation” [4]. Physiologic changes such as increased cardiac output and preload impact pulmonary blood flow as well as left- and right-sided heart filling pressures. Increased left ventricular (LV) filling pressures raise mean pulmonary artery pressure, create pulmonary venous hypertension, and exacerbate pulmonary hypertension in people with susceptible factors, such as previous porto-pulmonary hypertension. Right atrial and mean pulmonary artery pressure is more than twice as high in individuals with high cardiac output as it is in those with typical cardiac output [5]. Prolonged exposure to these hemodynamic alterations causes right-sided chamber enlargement and right heart failure (HF), and CCM patients may also present with right HF and changes in LV shape and function.

Right ventricular (RV) dysfunction has been linked to the prognosis of cirrhotic patients; many of these individuals exhibit indications of RV dysfunction [6]. As a result, assessing RV and LV function in individuals with cirrhosis is crucial [7, 8]. The majority of investigations examining the changes in right heart function in cirrhotic people have focused on classic right ventricular dysfunction indicators [9]. The right ventricle, on the other hand, resembles a pyramid, and measures such as Tricuspid Annular Plane Systolic Excursion (TAPSE), Fractional Area Change (FAC), and speckle tracking-derived Global Longitudinal Strain (GLS) provide information on the longitudinal movement of the free wall of the right ventricle. These conventional measures, once again, ignore the right ventricle’s trigon feature. Recent studies showed that the contractile properties of the right ventricular outflow tract (RVOT) play an important role in right ventricular activities [10, 11]. The Right Ventricular Outflow Tract-Systolic Excursion (RVOT-SE) is an essential RVOT systolic function metric [12]. There is yet to be a published study on RVOT function in cirrhotic patients. In this study, we evaluated the relationship between the severity of the disease and the RVOT-SE values in cirrhotic patients.

## 2. Materials and methods

### 2.1. Study design and population

Our retrospective cross-sectional study was in accordance with the Helsinki Declaration and was authorized by the local ethics committee (decision no: 2022/188, date: 05/07/2022).

In this single-center retrospective study, we screened 113 echocardiography records in patients with cirrhosis who were referred to the Cardiology Department’s echocardiography unit from the gastrointestinal and internal medicine departments between June 2018 and January 2022. When repeated procedures for the same patient were extracted, 95 echocardiography records were obtained. The study excluded 12 patients due to insufficient imaging quality, and 14 patients were not included because of having exclusion criteria.

Patients with organic valvular heart disease, coronary artery disease, connective tissue disorders, HIV, LV (left ventricular) systolic or diastolic (>grade II) dysfunction, chronic lung disorder, and atrial fibrillation were excluded from the study.

**Figure 1:**
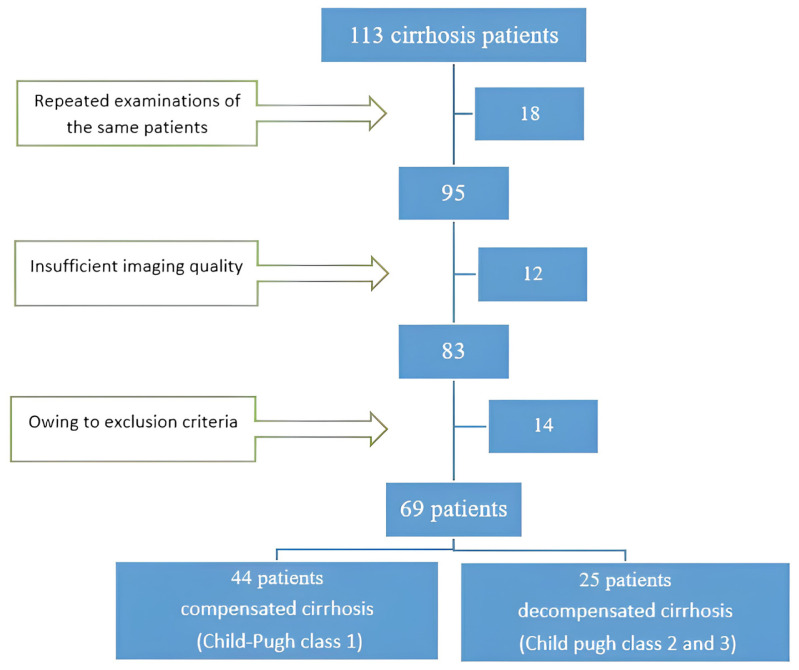
Selection of the study population.

Detailed medical histories were obtained in every case under investigation, emphasizing a complete history of liver cirrhosis, including etiology and prognosis. An extensive physical examination was performed on all patients, with attention paid to clubbing, ascites, spider nevi, lower limb edema, palmer erythema, and liver/spleen examinations. A 12-lead resting ECG was performed on all patients to detect velocity, rhythm, abnormal findings, and arrhythmias. For all patients, laboratory tests included a complete blood count, serum liver function tests such as aspartate aminotransferase (AST), alanine aminotransferase (ALT), serum albumin, serum bilirubin levels, prothrombin time, and international normalized ratio. According to the Child-Turcotte-Pugh score, liver cirrhosis is classified into A, B, or C categories based on bilirubin levels, prothrombin time, albumin, and encephalopathy severity [3]. So, there were 44 patients in Child-Pugh Class A (63.7%), 22 patients in Child-Pugh Class B (31.8%), and 3 patients in Child-Pugh Class C (4.3%). Afterward, the patients were separated into two groups: those with compensated cirrhosis (Child-Pugh class 1) and those with decompensated cirrhosis (Child-Pugh class 2 and 3). The study’s flowchart is presented in Figure 1.

### 2.2. Transthoracic echocardiography

The transthoracic echocardiogram was performed using the GE-Vingmed Vivid 7 System (GE-Vingmed Ultrasound, Horten, Norway) with a matrix probe. Obtained images were recorded at the echo Pac version 8.0 GE Healthcare. The echocardiographic parameters were obtained from parasternal and apical windows. At the end-exhale phase, three cardiac cycles were seen. The entire dataset was transferred to a workstation for offline processing (EchoPAC PC; GE Vingmed Ultrasound AS). The procedures outlined in an American Society of Echocardiography recommendation were used to perform traditional two-dimensional (2D) echocardiographic tests [13]. The LV ejection fraction (EF) was computed by using the biplane Simpsons technique. TAPSE was calculated by measuring the systolic displacement of the M mode longitudinally over the tricuspid annular plane and parallel to the right ventricle’s lateral wall. The E/A ratio was computed using pulsed-wave Doppler imaging of the mitral inflow profile to quantify the ventricular filling velocities in the early (E) and late (A) waves. All velocities were determined using a tissue Doppler imaging (TDI) sample volume located at the septal and lateral mitral annuli.

**Figure 2:**
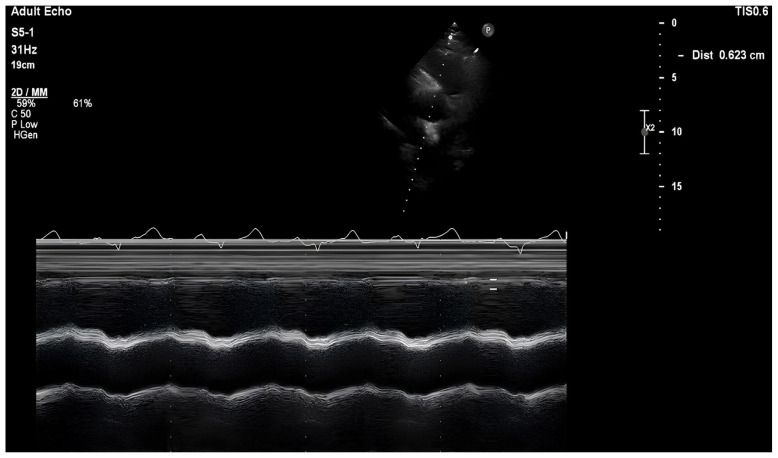
The RVOT-SE measurement in a patient with advanced cirrhosis.

RVOT-SE was determined utilizing enlarged cine-loops of the RVOT area acquired from the parasternal short-axis viewpoint using M-mode echocardiography. After the previously indicated adjustments to the focus, gain, and compression settings, imaging was performed at the level of the aortic valve at the maximum RVOT diameter with the ultrasound beam perpendicular to the RVOT walls [12]. Averaging three measurements supplied each data point. Figure 2 depicts an RVOT-SE depiction. The studies were carried out in each case by two skilled sonographers blinded to the results of the other test. The two studies were interpreted by two expert echocardiographers for each patient, and their judgments were likewise blinded. Intraclass correlation coefficients (ICCs) with confidence intervals were obtained using the mean squares from the ANOVA model to examine interobserver reliability between sonographers. The ICC evaluates rating reliability by comparing the variability of various ratings of the same subject to the total variance of all ratings and all subjects.

### 2.3. Statistical methods

The information was gathered, collated, and statistically examined. The Shapiro-Wilk test was used to assess the distributional features of the data before statistical analysis. Continuous variables were reported as mean ± standard deviation for normally distributed data, and group comparison was done using an independent two samples t-test. Nonnormally distributed data were reported as median (min.–max.), and the Mann-Whitney U test was used to compare groups. The frequency and percentage of categorical variables were reported, and the χ^2^ test was utilized for bivariate comparison. The logistic regression analysis methods with a single and multiple explanatory variable(s) were used. We evaluated the odds ratios with 95% confidence intervals for each research variable in the single explanatory variable logistic regression analysis, and the significance level of each factor/covariate was established. The original model, which included all significant independent variables, was fit in the multiple explanatory variable logistic regression analysis. The model was then evaluated for potential confounding effects using a backward-elimination strategy in a multiple explanatory variable logistic regression model. The factors/covariates were eliminated one at a time in this model, beginning with the factor/covariate with the highest significant p-value and continuing until all remaining factors had a two-sided p-value of 0.05. To determine the quality of fit, the Hosmer-Lemeshow test was used. The result was considered significant if the p-value was less than 0.05. SPSS 21.0 was utilized for all the statistical works. We performed this study with 69 patients, and post hoc power analysis (G Power 3.1.9.7) according to RVOT-SE measure with 69 patients was performed; power = 0.93, the effect size was 0.805.

## 3. Results

The research included 69 cirrhotic individuals. Table 1 shows the clinical characteristics of all cirrhotic individuals. When the patients’ Child scores were reviewed, 44 were categorized as Child-Pugh Class A (63.7%), 22 as Child-Pugh Class B (31.8%), and 3 as Child-Pugh Class C (4.3%). All patients were separated into two groups: those with compensated cirrhosis (Child-Pugh class 1) and those with decompensated cirrhosis (Child-Pugh class 2 and 3).

The patients’ demographic characteristics, medical history, cirrhosis complications, and laboratory data are summarized in Table 2. In terms of demographic and medical history features, neither group differed significantly. There were also no distinct pharmacological features. Serum albumin and sodium levels were considerably lower in group 2, but the prothrombin time was longer. The distribution of bilirubin, AST, ALT, platelet count, blood urea nitrogen, and creatinine was nonnormal, and group comparison using the Mann-Whitney U test revealed statistical significance for bilirubin, AST, and blood urea nitrogen. Group 2 had considerably higher medians for bilirubin, AST, and blood urea nitrogen. Other laboratory findings for each group were quite similar.

**Figure 3:**
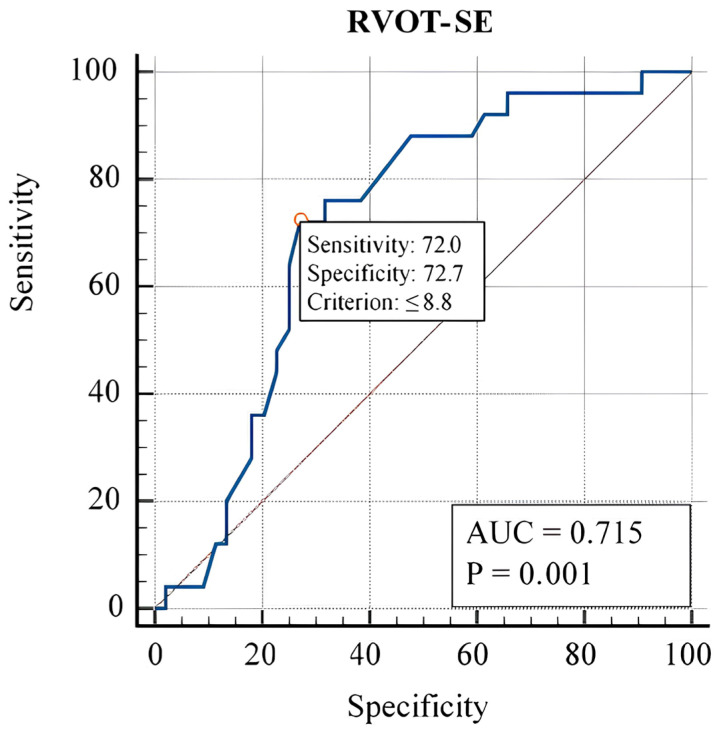
In the ROC analysis, RVOT-SE 8.8 cm/s predicted severe cirrhosis with 72% sensitivity and 72.7% specificity (AUC = 0.715, p < 0.001)

We revealed no statistically significant differences between groups in various standard echocardiographic tests that assessed the dimensions and function of the left ventricle (Table 3). There was no substantial difference in the E/e’ values obtained in the patient groups for filling pressures (6.771.85 vs. 7.482.80, p = 0.212). Nevertheless, a statistically significant difference in Right Ventricle Wall (RVW) (p = 0.014), systolic pulmonary artery pressure (sPAP) (p = 0.034), RVOT-SE (p = 0.003), and TAPSE (p = 0.042) were detected across cirrhosis groups (Table 3). Group 2 had a lower mean for all of these factors (Table 3). Intraclass correlation coefficients (ICCs) with confidence intervals were obtained using mean squares from an analysis of variance (ANOVA) model to examine interobserver reliability between sonographers. The ICC evaluates rating reliability by comparing the variability of various ratings of the same subject to the total variance of all ratings and all subjects. For echocardiographic variables, power analysis results ranged from 82% to 95%. RVW, sPAP, RVOT-SE, and TAPSE were statistically significant in a single explanatory logistic regression analysis of right ventricular echocardiographic variables. Next, using the backward elimination approach, a multiple explanatory variable logistic regression analysis research was undertaken for these four variables, and the findings suggest that RVOT-SE has a positive connection with cirrhosis severity (OR: 0.607; 95% CI: 0.425– 0.866; p = 0.006) (Table 4). According to the ROC curve study, RVOT-SE 8.8 cm/s predicted compensated-decompensated cirrhosis with 72% sensitivity and 72.7% specificity (AUC = 0.715, p < 0.001) (Figure 3).

## 4. Discussion

In the presented study, we mainly determined that; the RVOT-SE could predict decompensated cirrhosis reliably; also, RVOT-SE may be disturbed before the traditional RV systolic parameters. Moreover, RVW and sPAP were higher in more severe cirrhotic patients, but TAPSE was lower.

Cirrhosis has previously been demonstrated to affect left ventricular function, and numerous echocardiographic predictions have been linked to the severity of cirrhosis [6, 14–16]. Cirrhotic individuals may suffer right cardiac dysfunction, according to recent research [9,16,17]. In prior research, conventional right ventricular functional characteristics were employed. The majority of the measures emphasized in the study are longitudinal functional indices assessed over the right ventricular free wall and tricuspid annulus (such as TAPSE and St). The right ventricle’s complicated structure indicated some functional variations as well. When examining right ventricular function, the outflow tract in particular is frequently overlooked. Many congenital and acquired disorders, including ASD, Eisenmenger syndrome, TOF, APE, and inferior acute myocardial infarction, have been demonstrated to impair the function of the RVOT [11, 18–20]. According to the findings of these investigations, RVOT performance is critical in determining overall RV function.

Patients with liver cirrhosis have been known to have cardiac dysfunction since the 1960s. Cirrhotic cardiomyopathy refers to a group of cardiac abnormalities caused by liver cirrhosis. CCM symptoms include systolic dysfunction, diastolic dysfunction, a prolonged Q-T interval, and other electromechanical anomalies [21]. Hyperdynamic circulation, which is characterized by high cardiac output and increased cardiac effort, is a feature of advanced cirrhosis. Nevertheless, this may be clinically latent and may arise under physical or pharmacological stress due to a decreased afterload (reduced systemic vascular resistance) [14,15]. The majority of research on cirrhotic patients has been studied on LV function, which is commonly measured using tissue Doppler echocardiography [22]. Yet, in these people, cardiac dysfunction is frequently overlooked or detected after overt cardiac failure has occurred [6]. Right ventricular (RV) dysfunction has been connected to the prognosis of cirrhotic patients; many of these individuals exhibit indications of RV dysfunction [7]. As a result, assessing RV and LV function in cirrhotic patients is critical [6, 8]. Cirrhotic patients have increased right cardiac preload due to impaired liver function. Elevated hepatic venous pressures can affect RV function by increasing preload. The RV function is more difficult to evaluate than the LV function.

Cirrhotic individuals had worse diastolic and systolic left ventricular performance, according to previous research [6, 16, 22]. Modern speckle-tracking echocardiographic technologies have been demonstrated to detect these deteriorations in the preclinical period. In this disorder, which affects both the right and left ventricles, speckle-tracking echocardiographic techniques have been shown to impair right ventricular systole and diastole functions (in both clinical and subclinical phases) [9, 23–26]. Nevertheless, the methodologies used must allow for the capture of a snapshot to indicate how much RVOT is damaged and how it may predict the severity of the situation. RVOT-SE is the most important parameter that has lately appeared in assessing RVOT functions. RVOT-SE is calculated using the M-mode approach using parasternal short-axis echocardiographic images. To quantify right heart functions in cirrhotic patients, metrics based on the longitudinal motions of the free wall of the right ventricle, such as TAPSE, FAC, and right ventricular strain echo parameters, were frequently examined [27–30]. We hypothesized that RVOT functions would be affected early in cirrhotic patients by showing that disease severity could compromise RVOT functions, even when TAPSE is normal in cirrhotic cardiomyopathy patients. Given that cirrhotic patients’ pulmonary pressures rise gradually over time and clinical conditions associated with pulmonary hypertension, such as porto-pulmonary syndrome, early systolic dysfunction begins in the RVOT region, as the RVOT is the right ventricular region where increased pulmonary resistance and pressure are the first to act [31–33].

We anticipated that RVOT-SE, an indication of RVOT functions, might discriminate decompensated patients from compensated patients sooner than traditionally utilized RV systole measures in the current study. This idea, however, will need to be investigated and proven in bigger patient groups in the future. Comparative investigations using cardiac magnetic resonance imaging (CMRI) can be useful in this regard.

There are various limitations to our study. Firstly, it was not a multicenter research, and additional centers would need to confirm the illuminating results. Secondly, cardiac magnetic resonance (CMR), recognized as the gold standard modality for the study of ventricular function, was not employed since the CMR apparatus was not available at our center. Nevertheless, this study is the first to highlight a significant association between RVOT-SE and cirrhosis, providing a foundation for future investigations. Statements from the Introduction and Results sections should not be repeated here. The final paragraph should highlight the main conclusions of the study. The Results and Discussion sections may be combined.

In the presented study, we demonstrated that RVOT-SE could be associated with the severity of cirrhosis. The studies will enlighten the hypothesis, including larger patient cohorts and multicenters.

## Figures and Tables

**Table 1:** Clinical characteristics of all patients with cirrhosis.

	n = 69%
**Cause of liver cirrhosis, %**	
Hepatitis B	28 (40.6)
Hepatitis C	16 (23.2)
Alcohol	5 (7.2)
Wilson’s	4 (5.8)
Autoimmune liver disease	2 (2.9)
Primary biliary cirrhosis	3 (4.3)
Cryptogenic	3 (4.3)
Hepatic steatosis	2 (2.9)
Hemochromatosis	1 (1.4)
Budd Chiari Syndrome	1 (1.4)
Portal vein thrombosis	1 (1.4)
Others	3 (4.3)
**Complications of cirrhosis, %**	
Ascites	28 (40.6)
Splenomegaly	52 (75.4)
Varices	46 (66.7)
Variceal bleeding	14 (20.3)
Liver cancer	4 (5.8)
**MELD-Na Score**	**11.30 ± 4.29**
**Child-Pugh Class, %**	
A	44 (63.8)
B	21 (30.4)
C	4 (5.8)

**Table 2:** Baseline characteristics of the study groups.

Parameters	Group 1 (n = 44)	Group 2 (n=25)	P value
**Demographic:**			
Sex, male/femaleα	26/18	11/14	0.227
Age, year β	56.38 ± 12.75	56.36 ± 13.73	0.994
**Medical history:**			
Hypertensionα	12 (27.2%)	10 (40.0%)	0.276
Diabetes mellitusα	19 (43.2%)	12 (48.0%)	0.699
CKDα	2 (4.5%)	2 (8.0%)	0.555
**Complications of cirrhosis:**			
Ascitesα	6 (13.6%)	22 (88.0%)	**<0.001**
Varicesα	26 (59.1%)	20 (80.0%)	0.077
Variceal bleedingα	6 (13.6%)	8 (32.0%)	0.068
Splenomegalyα	35 (79.5%)	17 (68.0%)	0.285
Liver Cancerα	2 (4.5%)	2 (8.0%)	0.555
**Laboratory:**			
Albumin, g/L β	4.12 ± 0.51	3.10 ± 0.80	**<0.001**
Total bilirubin, mg/dL γ	0.96 (0.31–2.45)	2.15 (0.48–17.38)	**<0.001**
AST, U/L γ	26 (12–187)	50 (17–241)	**0.001**
ALT, U/L γ	22 (6–148)	29 (9–72)	0.083
Prothrombin time, INR β	1.24 ± 0.27	1.52 ± 0.62	**0.010**
Platelet count, cells/μL γ	98.75 (37–319)	104 (53.4–213)	0.803
Sodium, mmol/L β	138.61 ± 2.85	136.50 ± 4.20	**0.019**
Potasium, mmol/L β	4.28 ± 0.33	4.33 ± 0.40	0.540
Blood urea nitrogen, mg/dL γ	28 (13–92)	36 (16–152)	**0.026**
Creatinine, mg/dL γ	0.78 (0.48–2.63)	0.85 (0.56–1.73)	0.389
**Use of medications:**			
Beta Blockerα	21 (47.7%)	11 (44.0%)	0.765
Mineralocorticoid receptor antagonistα	10 (22.7%)	5 (20.0%)	0.792
Diureticα	17 (38.6%)	14(56.0%)	0.163

CKD: Chronic kidney disease, AST: Aspartate aminotransferase, ALT: Alanine aminotransferase, INR: International normalized ratio

α Categorical variables were reported as frequency (percent), and bi-variable comparison was conducted via Pearson’s chi-square test.

β Data were expressed as mean ± sd and compared using an independent two samples t-test.

γ Data were expressed as median (minimum–maximum) for nonnormally distributed continuous variables, and the p value was obtained using the Mann-Whitney U test.

Bold value indicates statistical significance at the p < 0.05 level.

**Table 3:** Echocardiography findings of patients. Data were expressed as mean ± sd and compared using independent two samples t-test

	Group 1 (n = 44)	Group 2 (n = 25)	p value
**LVEF**	61.77 ± 5.24	60.96 ± 4.43	0.516
**LA**	3.50 ± 0.29	3.61 ± 0.37	0.178
**LVED**	4.53 ± 0.33	4.49 ± 0.33	0.671
**LVES**	2.78 ± 0.45	2.68 ± 0.53	0.373
**IVS**	1.03 ± 0.14	1.08 ± 0.13	0.136
**PW**	1.19 ± 0.10	1.08 ± 0.10	0.617
**RVW**	6.81 ± 1.01	7.54 ± 1.38	**0.014**
**Mitral E**	82.43 ± 13.32	85.08 ± 21.13	0.525
**Mitral A**	80.22 ± 19.03	78.88 ± 16.30	0.767
**E’**	11.75 ± 2.35	11.14 ± 1.64	0.258
**A’**	13.11 ± 2.50	12.89 ± 1.70	0.708
**E/e’**	6.77 ± 1.85	7.48 ± 2.80	0.212
**sPAP**	23.70 ± 4.15	26.60 ± 7.01	**0.034**
**RVOT-SE**	9.59 ± 1.68	8.39 ± 1.27	**0.003**
**TAPSE**	24.62 ± 2.39	23.38 ± 2.39	**0.042**

LVEF: Left Ventricular Ejection Fraction, LA: Left Atrium, LVED: Left Ventricular End-Diastolic Diameter, LVESD: Left Ventricular End-Systolic Diameter, IVS: Interventricular Septum, PW: Posterior Wall, RVW: Right Ventricular Wall, Mitral E: Early Ventricular Filling Velocity, Mitral A: Late Ventricular Filling Velocity, E’: Ventricular Tissue Doppler Early Diastolic Velocity, A’: Ventricular Tissue Doppler Late Diastolic Velocity, sPAP: Systolic Pulmonary Artery Pressure, RVOT-SE: Right Ventricular Out Tract Systolic Excursion, TAPSE: Tricuspid Annular Plane Systolic Excursion.

Bold value indicates statistical significance at the p < 0.05 level.

**Table 4:** The result of multivariate logistic regression analysis for the prediction of decompensated cirrhosis.

	Univariate		Multivariate	
	OR (95% CI	p value	OR (95% CI)	p value
RVW	1.718 (1.088–2.713)	0.020	1.532 (0.947–2.479)	0.082
sPAP	1.104 (1.002–1.215)	0.045	1.107 (0.996–1.231)	0.058
RVOT-SE	0.603 (0.412–0.843)	0.005	**0.603 (0.412–0.843)**	**0.005**
TAPSE	0.794 (0.632–0.997)	0.047	0.876 (0.682–1.124)	0.298

RW: Right Ventricular Wall, sPAP: Systolic Pulmonary Artery Pressure, RVOT-SE: Right Ventricular Outflow Tract Systolic Excursion, TAPSE: Tricuspid Annular Plane Systolic Excursion.
